# Presynaptic muscarinic acetylcholine autoreceptors (M_1_, M_2_ and M_4_ subtypes), adenosine receptors (A_1_ and A_2A_) and tropomyosin-related kinase B receptor (TrkB) modulate the developmental synapse elimination process at the neuromuscular junction

**DOI:** 10.1186/s13041-016-0248-9

**Published:** 2016-06-23

**Authors:** Laura Nadal, Neus Garcia, Erica Hurtado, Anna Simó, Marta Tomàs, Maria A. Lanuza, Manel Santafé, Josep Tomàs

**Affiliations:** Unitat d’Histologia i Neurobiologia (UHN): Facultat de Medicina i Ciències de la Salut, Universitat Rovira i Virgili, Carrer St Llorenç num 21, 43201 Reus, Spain

**Keywords:** Motor end-plate, Motor nerve terminal, Cholinergic synapses, Neuromuscular junction

## Abstract

**Background:**

The development of the nervous system involves an initially exuberant production of neurons that make an excessive number of synaptic contacts. The initial overproduction of synapses promotes connectivity. Hebbian competition between axons with different activities (the least active are punished) leads to the loss of roughly half of the overproduced elements and this refines connectivity and increases specificity. The neuromuscular junction is innervated by a single axon at the end of the synapse elimination process and, because of its relative simplicity, has long been used as a model for studying the general principles of synapse development. The involvement of the presynaptic muscarinic ACh autoreceptors may allow for the direct competitive interaction between nerve endings through differential activity-dependent acetylcholine release in the synaptic cleft. Then, the most active ending may directly punish the less active ones. Our previous results indicate the existence in the weakest axons on the polyinnervated neonatal NMJ of an ACh release inhibition mechanism based on mAChR coupled to protein kinase C and voltage-dependent calcium channels. We suggest that this mechanism plays a role in the elimination of redundant neonatal synapses.

**Results:**

Here we used confocal microscopy and quantitative morphological analysis to count the number of brightly fluorescent axons per endplate in P7, P9 and P15 transgenic B6.Cg-Tg (Thy1-YFP)16 Jrs/J mice. We investigate the involvement of individual mAChR M_1_-, M_2_- and M_4_-subtypes in the control of axonal elimination after the *Levator auris longus* muscle had been exposed to agonist and antagonist *in vivo*. We also analysed the role of adenosine receptor subtypes (A_1_ and A_2A_) and the tropomyosin-related kinase B receptor. The data show that postnatal axonal elimination is a regulated multireceptor mechanism that guaranteed the monoinnervation of the neuromuscular synapses.

**Conclusion:**

The three receptor sets considered (mAChR, AR and TrkB receptors) intervene in modulating the conditions of the competition between nerve endings, possibly helping to determine the winner or the lossers but, thereafter, the final elimination would occur with some autonomy and independently of postsynaptic maturation.

## Background

The development of the nervous system involves the initial overproduction of synapses, which promotes connectivity, and a subsequent activity-dependent reduction in the number of synapses. This refines connectivity and increases specificity. Hebbian competition between axons with different activities (the least active are eliminated) seems to be a characteristic of the process and leads to the loss of roughly half of the overproduced elements and the functional consolidation of the remaining synapses in the adult [1, 2]. Synaptic contacts are lost throughout the nervous system during histogenesis [3, 4]. In newborn animals, the skeletal muscle fibers are polyinnervated by several motor axons [5] but at the end of the axonal competition, the endplates are innervated by a single axon. Because of its relative simplicity, the neuromuscular junction (NMJ) has long been used as a model for studying the general principles of synapse development in an attempt to understand the synapse elimination process [2, 6–12].

Various presynaptic receptors seem to play an important role in the axonal competition leading to synapse loss in the NMJ. The involvement of muscarinic acetylcholine autoreceptors (mAChRs) in the elimination process may allow direct competitive interaction between nerve endings through a differential activity-dependent acetylcholine (ACh) release. Then, the more active ending may directly punish those that are less active or reward themselves if the suitable mAChR subtypes are present in the competing axons. Our previous results indicate that, in postnatal muscles, there is an ACh release inhibition mechanism based on mAChR coupled to a PKC-VDCC intracellular cascade. In certain weak motor axons, this mechanism can depress ACh release and even disconnect synapses [13–17]. We suggest that this mechanism plays a central role in the elimination of redundant neonatal synapses because functional axonal withdrawal can indeed be reversed by mAChR, protein kinase C (PKC) or voltage-dependent calcium channels (VDCC) block [17, 18]. However, local differential effectiveness and differential activity will determine eventual success, since an axon that fails at one synapse (muscle cell) may be successful at another [19], which suggests complex regulation involving other receptors and postsynaptic- (and glial cell) derived factors. Both neurotrophin receptors (NTR) and adenosine receptors (AR) belong to leading presynaptic signalling pathways. In the adult NMJ, the activity of one of these receptors can modulate a given combination of spontaneous, evoked and activity-dependent release conditions and a close dependence between them exist [20]. These receptors and their intracellular signalling may help to refine the molecular and structural organization of the newborn synapses so that they can acquire their mature form.

Here we investigate the involvement of individual mAChR subtypes in the control of synapse elimination. We also analyse the role of AR (A_1_ and A_2A_) and trompomyosin-related kinase B receptor (TrkB). The data show that the three receptor sets considered cooperate in the elimination process and promote axonal disconnection at the beginning of the second postnatal week independently of the postsynaptic maturation of the nicotinic receptor cluster.

## Results

### Postnatal elimination of nerve terminals

#### Normal evolution of postnatal polyneuronal innervation in the NMJ

**Fig. 1:**
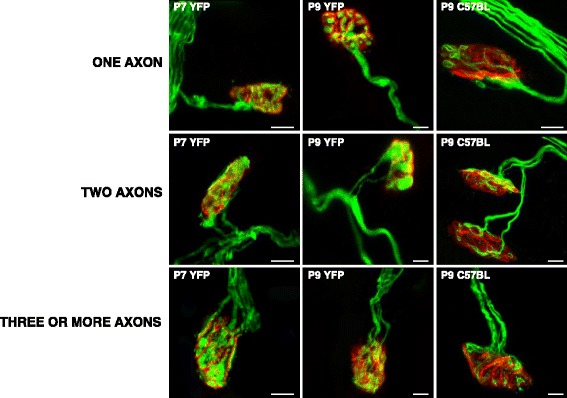
The picture shows some representative confocal immunofluorescence images of the singly- and polyinnervated NMJ from YFP and C57BL/J6 mice. Scale bar: 10 μm

**Fig. 2:**
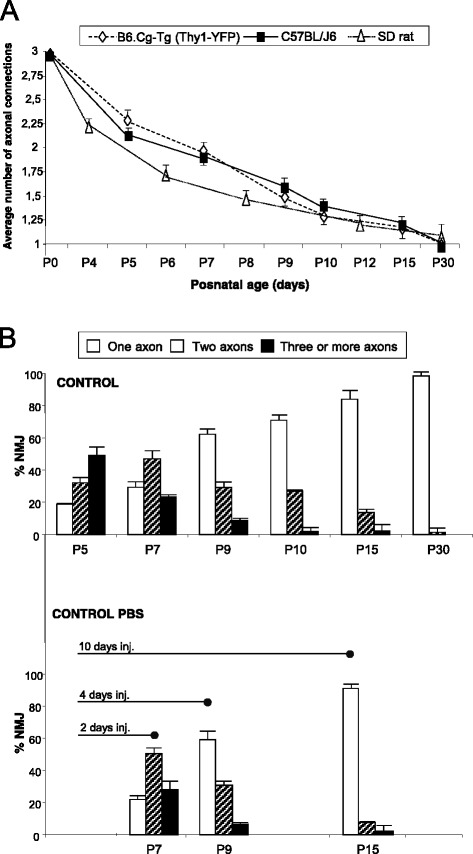
Postnatal evolution of polyneuronal innervation. In **a**, comparison of the results of axon counts in fluorencence immunohistochemistry LAL preparations of YFP and C57BL/J6 mice. The histogram in **b** shows the percentage of singly-, dually- and triply- (or more) innervated synapses in YFP animals on the postnatal days studied without any experimental manipulation (control non-PBS, without subcutaneous injection), and also at P7, P9 and P15 after two (days 5–6), four (days 5–8) and ten (days 5–14) daily subcutaneous PBS applications respectively (control PBS). No differences are observed between PBS and non-PBS preparations (Fisher’s test: *p >* 0,05)

Figure 1 shows some representative confocal immunofluorescence images of singly- and polyinnervated NMJs from YFP (autofluorescent axons) and C57BL/J6 (axons stained with anti neurofilament fluorescent antibody) mice. The images show that it is feasible to accurately count the number of axons in both preparations. Firstly, we investigated in our experimental model the normal postnatal elimination of the excess synaptic contacts. Figure 2a shows axonal counts in fluorescent immunohistochemistry LAL preparations (average number of axonal connections per NMJ) from B6.Cg-Tg (Thy1-YFP) – hereafter YFP – and C57BL/J6 mice. The figure also shows previous data [10, 21–23] from Sprague-Dawley (SD) rats to show similarities between rodents. The histogram in Fig. 2b shows the percentage of singly-, dually- and triply- (or more) innervated synapses in YFP for the postnatal days (P) considered without any experimental manipulation (control, non-PBS). Because the present work is based in subcutaneous injection procedure, we also wanted to control that subcutaneous injection by itself does not affect the elimination process in the control animals. Figure 2b also shows the percentage of singly-, dually- and triply- (or more) innervated synapses at P7 (*n* = 2315 NMJs, *N =* 10 mice), P9 (*n* = 2647 NMJs, *N =* 10 mice) and P15 (*n* = 1056 NMJs, *N =* 4 mice) after two (days 5, 6), four (days 5–8) and ten (days 5–14) subcutaneous PBS injections, respectively (control PBS). No significant differences are observed between PBS (the control for subcutaneous injections) and non-PBS (without subcutaneous injection) preparations ( *p* > 0,05, Fisher’s test).

#### Stimulation of the mAChR. Effect of oxotremorine

**Fig. 3:**
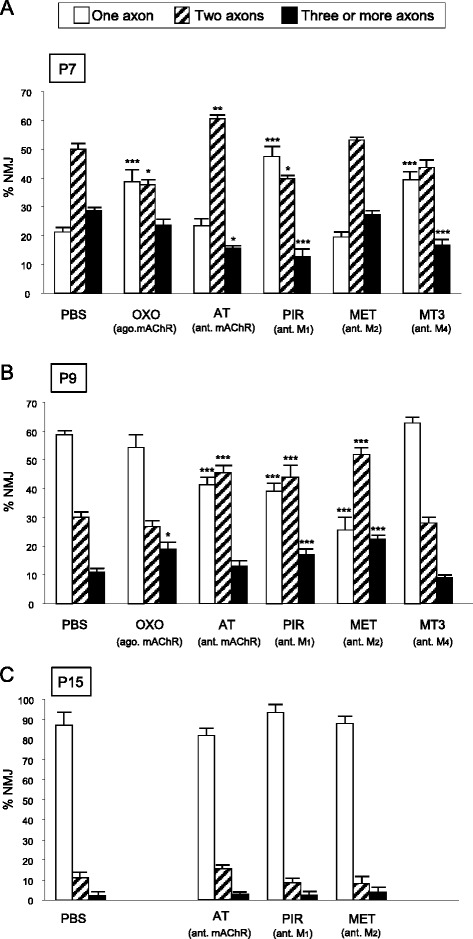
Changes in polyneuronal innervation of the NMJ after stimulation and inhibition of the mAChR. The figure shows the percentage of singly-, dually- and triply- (or more) innervated NMJs in the untreated YFP control mice (exposed to PBS applications) and after 2 (P7 in **a**), 4 (P9, in **b**) and in some cases 10 (P15, in **c**) applications (one application every day after P5) of the mAChR agonist (ago.) oxotremorine (OXO) and such antagonists (ant.) as atropine (AT), pirenzepine (PIR), methoctramine (MET) and muscarinic toxin 3 (MT3). Fisher’s test: * *p* < 0,05, ** *p* < 0,01, *** *p* < 0,005

Figure 3 shows the percentage of singly-, doubly- and triply (or more) innervated NMJs in the untreated YFP control mice and after 2 (P7), 4 (P9) and in some cases 10 (P15) applications (one application every day after P5) of the mAChR agonist oxotremorine (OXO) and such antagonists as atropine (AT), pirenzepine (PIR), methoctramine (MET) and muscarinic toxin 3 (MT3). We first used the potent and well characterized unselective agonist OXO. A subcutaneous application on the YFP LAL muscle surface every day (at P5 and P6) results in a significant acceleration at P7 of the axonal elimination process (Fig. 3a; Fisher’s test; *n* = 820 NMJs, *N =* 4 mice), because of the increase in monoinnervated NMJs (*p* < 0,005) and the reduction in dual (*p* < 0,05) synapses. It seems that the muscarinic mechanism, when stimulated, accelerates the axonal elimination rate and transition to the monoinnervation state.

However, four applications (P5-P8) of OXO (Fig. 3b; Fisher’s test; *n* = 865 NMJs, *N =* 4 mice) do not lead to any significant change at P9 (*p >* 0,05) in monoinnervated and dual junctions though a small increase of the fraction of synapses with three or more axons is observed (*p* < 0,05). This indicates that the effect of muscarinic stimulation diminishes and tends to peak close to the normal values of axonal elimination around four days after stimulation has begun. Therefore, there is a window around P5-P6 in which mAChR can be forced to accelerate synapse elimination. However, exogenous stimulation with the agonist reveals only that muscarinic signalling has the potential to accelerate postnatal axonal disconnection but does not explain what the tonic muscarinic control is like in a normal situation. Therefore, we investigate how blocking the M_1_, M_2_ and M_4_ mAChR subtypes *in toto* or selectively (those subtypes observed in functional developing NMJ, [13, 14, 24, 25]) can affect synapse elimination.

#### Unselective inhibition of mAChRs. Effect of atropine

Figure 3a shows that two subcutaneous applications of AT (at P5 and P6) in the YFP LAL muscles analysed at P7 significantly reduce the percentage of triple junctions (*p* < 0,05), increase the percentage of dual junctions (*p* < 0,01), and have no effect on the percentage of single junctions (Fisher’s test; *n* = 1343 NMJs, *N =* 3 mice). Thus, the rate of transition from three to two speeds up but the overall process does not continue to the point of significantly increasing monoinnervation. This indicates that AT has a dual effect: namely, it increases axon loss in triple junctions and reduces loss in double NMJs. It seems that NMJs or nerve terminals of different levels of maturity have different sensitivities and respond differently to this potent muscarinic pan-inhibitor.

Daily AT applications between P5 and P8 lead to a significant retardation of axonal elimination at P9 (Fig. 3b; Fisher’s test; *n* = 1032 NMJs, *N =* 4 mice) with persistent polyinnervation due to the higher percentage of dual junctions (*p* < 0,005) the corresponding decrease in monoinnervated synapses (*p* < 0,005) and an almost normal number of triple junctions (*p >* 0,05). This clearly indicates that blocking the mAChR can persistently obstruct the two-to-one transition of the elimination processs. However, unlike the OXO effect (which tends to disappear at P9 after accelerating elimination at P7), the effect of AT seems to be maintained throughout the period P5-P9 at least in relation to the two-to-one transition. It seems, then, that in normal conditions, the presynaptic muscarinic mechanism increases the rate of axonal loss at least in dual junctions in the period P5-P9 and that this effect can be increased at P7 by using an exogenous agonist.

#### Selective block of the mAChRs

How are the various mAChR subtypes that operate in the postnatal NMJ (M_1_, M_2_ and M_4_; [16]) involved in axonal elimination? We selectively blocked M_1_ (PIR), M_2_ (MET) and M_4_ (MT3) and observed the NMJ at P7 (daily applications on the LAL surface at P5 and P6, Fig. 3a) and P9 (applications between P5-P8, Fig. 3b). At P7 two subcutaneous PIR applications significantly reduced the percentage of triple (*p* < 0,005) and dual junctions (*p* < 0,05) and greatly increased the percentage of single junctions (*p* < 0,005, Fisher’s test; *n* = 915 NMJs, *N =* 4 mice). Thus, both the three-to-two and the two-to-one rates of transition accelerated considerably and the overall elimination process speeded up. This may indicate that in the normal situation the role of M_1_ is to slow elimination down because when it is uncoupled from PIR, the elimination process accelerates. Interestingly, the M_4_ blocker MT3 has almost exactly the same effect as the M_1_ blocker PIR (Fisher’s test; *n* = 895 NMJs, *N =* 4 mice), whereas the M_2_ blocker MET does not have a significant effect at P7 after the two subcutaneous applications (*p >* 0,05, Fisher’s test; *n* = 1012 NMJs, *N =* 4 mice). As an additional control, in P7 C57BL/J6 animals treated with MET we found the same result (Fisher’s test; Control PBS (*n* = 1533 NMJs, *N =* 6 mice): 1 axon: 22.69 % ± 1,04 % ; 2 axons: 50.20 % ± 2.75 % ; 3 or more axons: 27.11 % ± 3.18 %. MET application (*n* = 911 NMJs, *N =* 3 mice): 1 axon: 22.22 % ± 2.56 % (*p >* 0,05); 2 axons: 50.00 % ± 2.74 % (*p >* 0,05); 3 or more axons: 27.78 % ± 2.38 (*p >* 0,05)). Thus, at P7 the ensemble M_1_/M_4_ seems to be involved in a mechanism that delays elimination because when it is blocked the percentage of monoinnervated junctions increased and caused a fast three-to-one transition.

Nevertheless, how can it be explained that at this time (P7) the two-to-one transition is accelerated by the selective blockers PIR and MT3 (and not affected by MET), but that when all mAChR subtypes were blocked with AT this transition was partially delayed? Blocking the whole ensemble of subtypes with AT has a somehow diferent effect than the individual effects of mAChR subtypes. This apparent contradiction observed with the effects of selective and unselective pharmacological muscarinic inhibitory substances at P7 seems to suggest the existence of other confluent signalling pathways that take part in the process (see below).

However, daily applications of these substances for four days (P5-P8) lead to a much more clearly defined situation at P9 (Fig. 3b). As stated above, four AT applications delay elimination, maintain the number of dual junctions and decrease the number of singly-innervated NMJ, which indicates that the two-to-one transition is slowing down. The same effect (even greater because of the considerable delay in the three-to-two transition) is obtained by blocking M_1_ (PIR, *p* < 0,005, Fisher’s test; *n* = 1293 NMJs, *N =* 3 mice) and M_2_ (MET, *p* < 0,005, *n* = 976 NMJs, *N =* 4 mice) but not in this case with the M_4_ blocker MT3 (*p >* 0,05, *n* = 1177 NMJs, *N =* 4 mice). As an additional control, in P9 C57BL/J6 animals treated with MT3 we found the same result (Fisher’s test; Control PBS (*n* = 1352 NMJs, *N =* 5 mice): 1 axon: 48.17 % ± 4.54 % ; 2 axons: 36.73 % ± 2.76 % ; 3 or more axons: 15.10 % ± 4.97 %.MT3 aplications (*n* = 906 NMJs, *N =* 4 mice): 1 axon: 51.2 % ± 5.77 % (*p >* 0.05); 2 axons: 39.32 % ± 2.53 % (*p >* 0.05); 3 or more axons: 9.48 % ± 2.32 (*p >* 0.05)). These data indicate that at this point in the elimination process, both M_1_ and M_2_ subtypes cooperate in favouring the full sequence of synapse elimination.

To investigate the possible persistence of the mAChR effect throughout the period of synapse elimination, we made daily applications of AT (the unselective mAChR antagonist), PIR and MET (the M_1_ and M_2_ selective antagonists that are effective at modulating axonal elimination at P9) between P5 and P15 (in normal conditions almost 90% of NMJs were monoinnervated at P15). In spite of the continued presence of unselective and selective inhibitors, we found that the elimination process came to its normal conclusion by the end of the second postnatal week (Fig. 3c; (*p >* 0,05, Fisher’s test; AT: *n* = 720 NMJs, *N =* 3 mice; PIR: *n* = 924 NMJs, *N =* 3 mice; MET: *n* = 870 NMJs, *N =* 3 mice)). This reinforces the suggestion that several signalling mechanisms between the endings in competition cooperate (and substitute each other) to resolve the correct synaptic connection in a multifactorial process.

#### Other signalling mechanisms involved in axonal loss

**Fig. 4:**
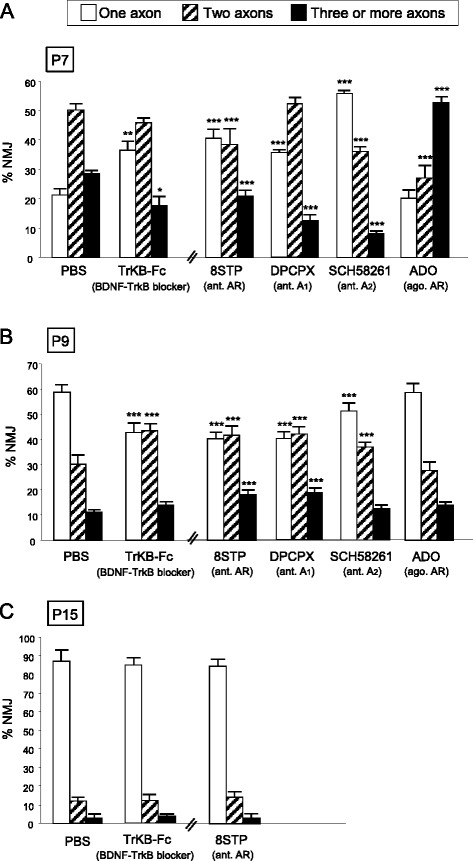
Involvement of adenosine receptors (AR) and TrkB receptors in axonal elimination. The figure shows the percentage of the singly-, dually- and triply- (or more) innervated NMJs in the YFP control mice exposed to PBS, and after 2 (P7 in **a**), 4 (P9, in **b**) and in some cases 10 (P15, in **c**) applications (one application every day after P5) of the BDNF-TrkB blocking pathway agent TrkB-Fc to sequester endogenous BDNF/NT-4 neurotrophins and the AR pan-inhibitor 8SPT and the AR agonist (ago.) ADO. We also studied axonal elimination after selectively blocking A_1_R with the antagonist (ant.) DPCPX and inhibiting A_2A_R with SCH-58261. The control for these selective inhibitors was PBS + DMSO (not shown in the figure) which shows no differences from PBS used as a control by itself. Fisher’s test * *p* < 0,05, ** *p* < 0,01, *** *p* < 0,005

Several signalling pathways connect the cells that make synapses. Here, we studied the possible involvement of adenosine receptors and neurotrophin receptors (here the representative TrkB receptor for brain-derived neurotrophic factor (BDNF) and neurotrophin-4 (NT-4) in the complex period of axonal elimination around P7-P9 (Fig. 4). To the LAL muscle, we subcutaneously applied the AR inhibitor 8SPT, the AR agonist ADO and the TrkB blocking pathway agent TrkB-Fc to sequester endogenous BDNF/NT-4 neurotrophins. With the 8SPT (*n* = 920 NMJs, *N =* 4 mice) and TrkB-Fc (*n* = 1113 NMJs, *N =* 4 mice) blockers at P7 we observed a clear acceleration in the three-to-two rate (Fisher’s test; 8SPT: *p* < 0,005; TrkB-Fc: *p* < 0,05) that was very similar to the acceleration in the two-to-one rate. These substances accelerate axonal elimination on the NMJ and, therefore, the physiological role in normal conditions of the AR and TrkB pathways at P7 seems to delay the axonal loss process. This is confirmed for the AR because exposure to the physiological agonist ADO results in a significantly higher number of triple junctions and a significant reduction in the number of dual junctions (*p* < 0,005, Fisher’s test; *n* = 923 NMJs, *N =* 4 mice). This indicates an ADO-induced retardation of axonal elimination. Which AR subtypes are involved in the ADO effect? We analysed axonal elimination after selectively blocking A_1_R with DPCPX (*n* = 1160 NMJs, *N =* 4 mice) or A_2A_R inhibition with SCH-58261(*n* = 963 NMJs, *N =* 4 mice) (Fig. 4a). The data show that axonal loss (the full three-to-one transition) is accelerated by both inhibitors (*p* < 0,005), which indicates that in normal conditions without inhibition both A_1_R and A_2A_R are associated with delaying loss.

Interestingly, at P9, neurotrophin signalling seems to reverse their coupling to the axonal loss process because TrkB-Fc (acting between P5-P8) considerably delays elimination (resulting in more dual and fewer monoinnervated NMJ; *p* < 0,005, Fisher’s test; *n* = 863 NMJs, *N =* 4 mice), which indicates that in a normal situation BDNF/NT-4 mediators change their role and accelerate elimination, as has been described above for the muscarinic mechanism. At P9, the purinergic mechanism also seems to tonically accelerate axonal elimination to the maximum rate because the AR pan-inhibitor 8SPT delays the process (an effect of the A_1_R and A_2A_R, Fig. 4b; Fisher’s test; *n* > 900 NMJs, *N =* 4 mice in each case) with no effect of the agonist ADO (*p >* 0,05, *n* = 908 NMJs, *N =* 4 mice).

Therefore, it seems that AR may behave biphasically in the critical period between 5-9 postnatal days. An initial delay in axonal loss at P7 (an A_1_R- and A_2A_R-mediated effect which can be reinforced by exogenously added ADO) is followed by an A_1_R- and A_2A_R-mediated tonic acceleration at P9.

To sum up, the two receptor sets (TrkB and AR) initially delay (P7) axonal loss but promote axonal disconnection at the beginning of the second postnatal week (P9) as mAChRs do. Figure 10 shows a graphic representation of these actions.

However, the experimental groups also differ with respect to the duration of receptor perturbation (two, four, and ten injections at P7, P9, and P15, respectively). Some effects may therefore be due to different durations of receptor inhibition. For instance, compensatory mechanisms may have more or less time to counteract receptor blockade. Thus, it is interesting to evaluate synapse development at different time points after inhibiting receptors for the same time. For the action of some blockers (MET, DPCPX and TrkB-Fc), we probe synapse development at P9 after inhibiting receptors for only two days (from P7-P9). The results show that the three blockers studied significantly reduce the percentatge of the monoinnervated junctions (with respect to the untreated control at P9) independently of their application during four or only two days before the observation at P9. This data reinforce the idea that the receptors role, in normal conditions without the inhibitors, can be to accelerate axonal loss. Interestingly, there are some differences between the blockers. The M_2_ blocker MET produces the same effect after four or two days (25.6 ± 1.04 % and 27.2 ± 1.1 % monoinnervated synapses respectively, Fisher’s test, *p >* 0,05). However, the A_1_ blocker DPCPX and the TrkB pathway blocker TrkB-Fc, significantly reduce the monoinnervated synapses even more after two days than after four days of application over the LAL muscle surface (DPCPX: two days 33.7 ± 1.16 %, four days 39.8 ± 1.09 %, *p* < 0,05; TrkB-Fc: two days 30.9 ± 1.12 %, four days 42.75 ± 1.07 %, Fisher’s test, *p* < 0,05). These data reinforce our interpretation that the M_2_ receptors start to accelerate axonal elimination around P7 whereas A_1_ and TrkB are involved in the initial delay (P5-P7) of axonal loss before shifting to promote axonal disconnection at the beginning of the second postnatal week (P9). The absence of the A_1_ and TrkB inhibition between P5-P7 results in a strong effect of the inhibitors when applied between P7-P9.

To assess the overall capacity of the considered signaling on axonal elimination, we investigate the overall effect of prolonged global receptor perturbation on axon number at P15. We studied axon number after more prolonged general block of mAChRs (AT, see above Fig. 3c), ARs (8SPT) and TrkBRs (TrkB-Fc) and found that in all cases, in spite of the continued presence of the inhibitors, monoinnervation is achieved in about 90 % of NMJ at P15 (Fig. 4c; (*p >* 0,05, Fisher’s test; TrkB-Fc: *n* = 825 NMJs, *N =* 3 mice; 8SPT: *n* = 720 NMJs, *N =* 3 mice)). We conclude that the modulation of axonal competition and the final process of axonal disconnection and loss seems differentially regulated.

Finally, we made some preliminary experiments to show a real cooperation between the receptors. For this purpose we selected receptors which perturbation produces a strong effect on axonal loss at P9 and applied simultaneously their inhibitors in a LAL muscle in four animals. We associated one AR blocker (DPCPX or SCH-58261) with the M_1_ blocker PIR. We found that both DPCPX and SCH-58261 add their individual delaying effect on axonal loss to the delaying effect of PIR resulting in 58 % and 36 % respectively less monoinnervated junctions that with PIR only (monoinnervated NMJ after PIR, 39 ± 1.1 %; after PIR + DPCPX, 16.4 ± 1.08 %; after PIR + SCH-58261, 24.8 ± 0.8 %. The two inhibitor associations differ significatively from the PIR only effect, *p* < 0,005).

### Postsynaptic receptors cluster during postnatal maturation

#### mAChR influence on the postsynaptic maturation

**Fig. 5:**
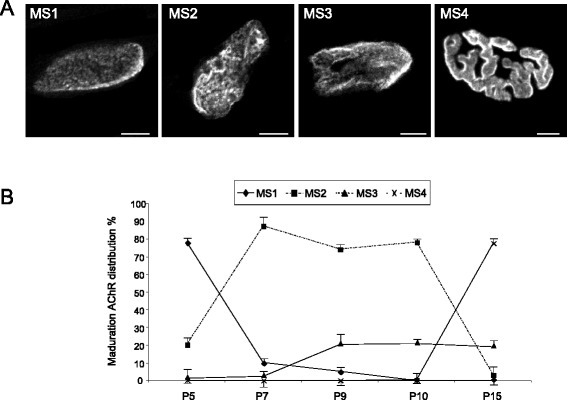
Postnatal morphological maturation of the postsynaptic apparatus. The axonal elimination process is accompanied by changes in the morphology of the nAChR clusters in the postsynaptic membrane. **a**, the following maturation stages (MS1–MS4) were defined. MS1: Uniform nAChR oval plaque with an indistinct boundary seen in the majority of NMJs at birth. A uniformly distributed porosity can be observed within this plaque. MS2: nAChR elongated oval plaque with a few hints of inhomogeneities in receptor density. The nAChRs are denser on a few narrow ridges that occur within the plaque. MS3: An oval nAChR plaque with one or more fluorescence-free “holes.” These holes are not innervated. MS4: The oval nAChR areas have been transformed into a more mature branched pattern with a moderately convoluted external border and high and low receptor density areas. The edge of the holes usually has a high density of receptors. Scale bar: 10 μm. **b, ** shows the percentages of the MS1-MS4 nAChR clusters plotted in the interval P5-P15 days

We analysed the morphological maturation of the postsynaptic apparatus in the same experimental conditions as those in the previous study on axon loss. The axonal elimination process is accompanied by changes in the morphology of the nicotinic ACh receptor (nAChR) clusters in the postsynaptic component. On the basis of criteria from previous studies on developing mammalian NMJs [23, 26–30], the following maturation stages (MS1–MS4) were defined (Fig. 5a). As normal maturation takes place, changes in the nAChR distribution transform the uniform nAChR oval plaque with an indistinct boundary seen at birth (MS1) into an elongated plaque with a few hints of heterogeneities in receptor density (MS2). This then changes into clusters with small areas of low nAChR density appearing as holes (MS3) that are not innervated. This morphology leads to an increasingly structured pattern of fluorescently labelled independent primary gutters (MS4) below the nerve terminals. Figure 5b shows the percentages of the MS1-MS4 nAChR clusters plotted at days P5-P15.

#### Stimulation and inhibition of the mAChRs

**Fig. 6:**
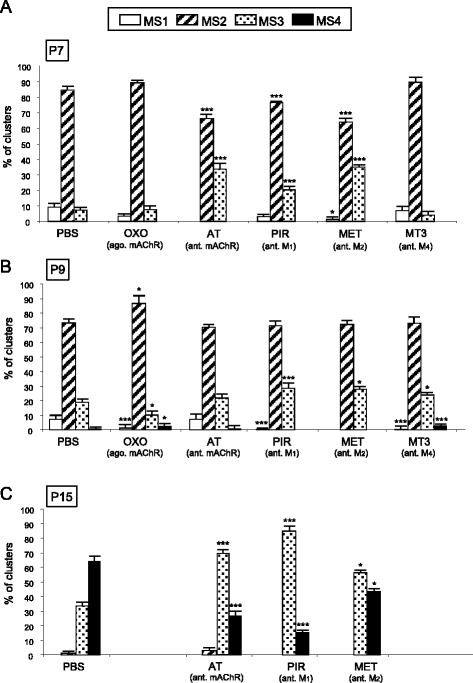
Maturation of postsynaptic nAChR clusters after stimulation and inhibition of mAChRs. Percentage of MS1-MS4 clusters in the NMJ of untreated YFP control mice (exposed to PBS), and after 2 (P7, in **a**), 4 (P9, in **b**) and in some cases 10 (P15 in **c**) applications of the muscarinic substances considered: OXO, AT, PIR, MET and MT3. Fisher’s test * *p* < 0,05, ** *p* < 0,01, *** *p* < 0,005

**Fig. 7:**
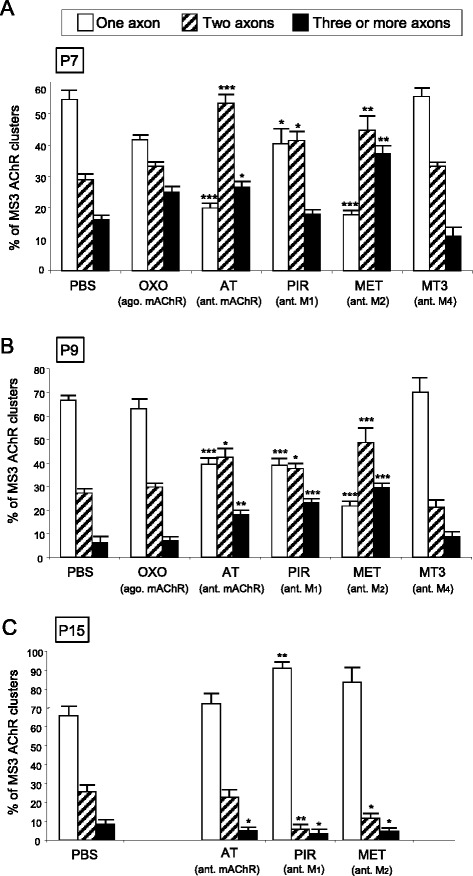
Pre- and postsynaptic maturation in the MS3 clusters after stimulation and inhibition of the mAChRs. For each day considered (P7 in **a**, P9 in **b** and P15 in **c**) the figure shows the percentage of MS3 clusters (the oval nAChR plaques with fluorescence-free holes that mature at a faster rate) with one, two and three or more axons as an indication of the appropiate correspondence of the pre- and postsynaptic maturation. Fisher’s test * *p* < 0,05, ** *p* < 0,01, *** *p* < 0,005

Figure 6 shows the percentage of MS1-MS4 clusters in the NMJ of the untreated YFP control mice (PBS) and after 2 (P7, Fig. 6a), 4 (P9, Fig. 6b) and 10 (P15, Fig. 6c) applications of the muscarinic substances considered. Figure 7a, b and c also show the percentage of MS3 clusters (postsynaptic clusters in advanced morphological maturation) with one, two or three (or more) axons for each day. This percentage can be taken as an indication of the correspondence between pre- and postsynaptic maturation. After the mAChR antagonists AT, PIR and MET (MT3 does not unambiguously modify the postsynaptic clusters) had been applied for two days, at P7 we found changes in the morphological maturation of the postsynaptic apparatus. Generally there was a high percentage of differentiated MS3 clusters (*p* < 0,005, Fisher’s test) and fewer MS1 and MS2 (*p* < 0,005) (Fig. 6a). Interestingly, many of these MS3 clusters are still innervated by 2–3 axons (Fig. 7a), which indicates some imbalance in the appropiate pre- and postsynaptic correspondence. The fact that postsynaptic maturation accelerates after muscarinic inhibition supports the notion that in normal conditions (without inhibition) the M_1_ and M_2_ subtypes have a tonic role and delay maturation. Because OXO does not have a definite significant effect (*p >* 0,05) (Fig. 6a), the tonic muscarinic effect may operate at close to its maximum rate.

At P9, the selective muscarinic drugs PIR, MET and MT3 accelerated cluster maturation, and produced fewer MS1 and more MS3 clusters (Fisher’s test; for MS3: PIR (*p* < 0,005); MET i MT3 (*p* < 0,05); even MS4 for MT3; see Fig. 6b), many of which (for AT, PIR and MET experiments) were innervated by 2–3 axons as they were at P7 (Fig. 7b). This also indicates that at P9 the M_1_, M_2_ and M_4_ subtypes are involved in delaying the normal maturation process in normal conditions. However, AT does not change the normal percentage of the cluster subtypes (though the MS3 subtype is also innervated by 2–3 axons, *p >* 0,05) and OXO moderately accelerates maturation (by reducing MS1 (*p* < 0,005) and increasing the MS2 subtype, *p* < 0,01). Thus, the use of the subtype-unselective drugs AT and OXO reveal the complex involvement of the mAChRs in the morphological maturation process of the postsynaptic receptor clusters. The coincident contribution of other signalling will be considered below.

With the unselective mAChR antagonist AT and the selective M_1_ and M_2_ muscarinic inhibitors, and specially with PIR, we observed at P15 that postsynaptic maturation seems to be slower and partially retained at the MS3 subtype (Fig. 6c) though most MS3 are already monoinnervated in the presence of AT, PIR and MET (Fig. 7c).

Thus, as far as postsynaptic clusters are concerned, in normal conditions mAChRs tend to produce some delay in maturation throughout the P5-P9 period and this effect is extended at P15 when axonal elimination is almost complete whether muscarinic modulators are used or not.

#### Other signalling mechanisms involved in postsynaptic maturation

**Fig. 8:**
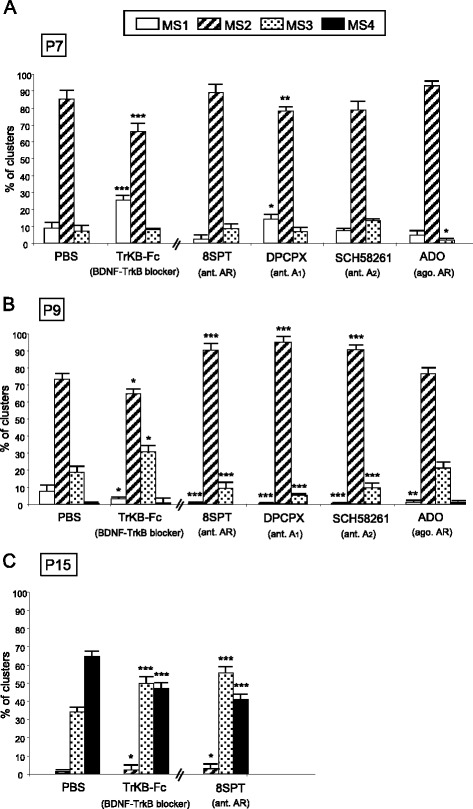
Involvement of the AR and TrkB receptors in the morphological maturation of the postsynaptic apparatus. The figure shows the percentage of the MS1-MS4 clusters in the NMJ of the untreated YFP control mice (exposed to PBS), and after 2 (P7 in **a**), 4 (P9, in **b**) and in some cases 10 (P15, in **c**) applications (one application every day after P5) of the TrkB blocking chimera TrkB-Fc, the AR pan-inhibitor 8SPT and the AR agonist ADO. We also studied axonal elimination after selectively blocking A_1_R with DPCPX and inhibiting A_2A_R with SCH-58261. Fisher’s test: * *p* < 0,05, ** *p* < 0,01, *** *p* < 0,005

**Fig. 9:**
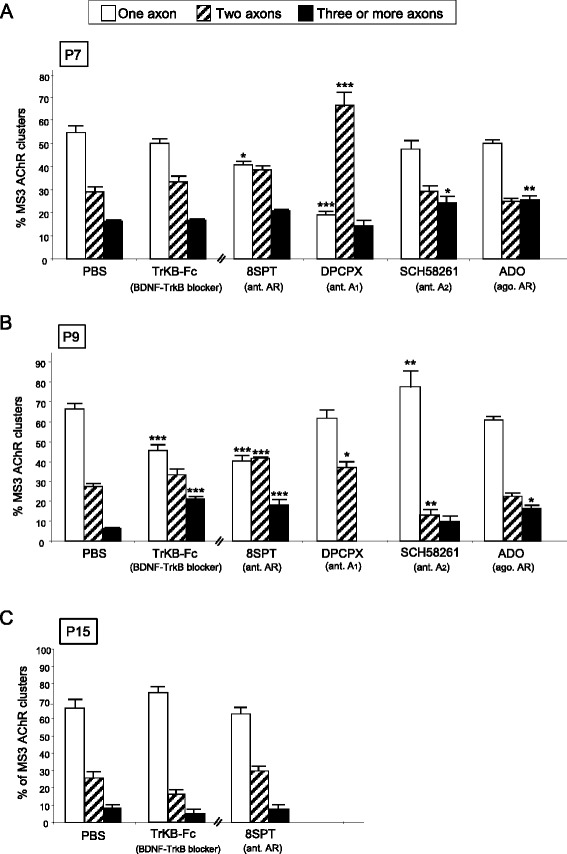
Pre- and postsynaptic maturation in the MS3 clusters. AR and TrkB pathways modification. For each day considered (P7 in **a**, P9 in **b** and P15 in **c**), the figure shows the percentage of MS3 clusters with one, two and three or more axons as an indication of the appropiate correspondence of the pre- and postsynaptic maturation. Fisher’s test: * *p* < 0,05, ** *p* < 0,01, *** *p* < 0,005

Figure 8a shows that after two days of using TrkB-Fc to sequester endogenous BDNF/NT-4, nAChR maturation is delayed at P7 because of the persistence of many MS1 clusters (*p* < 0,005, Fisher’s test). This indicates that the normal stimulation of the TrkB pathway promotes postsynaptic maturation at around P7. This tendency is reversed at P9 after four days of exposure to TrkB-Fc because of the clear increase in the MS3 subtype with respect to the untreated control (*p* < 0,05, Fig. 8b). In addition, many of these MS3 clusters are polyinnervated (with three or more axons, *p* < 0,005, Fig. 9b). Ten applications (one application every day after P5) of TrkB-Fc reveal some delay of the postsynaptic maturation at P15 (increased MS3 and less MS4 clusters, Fig. 8c). Thus, the TrkB pathway seems to have a complex effect on postsynaptic maturation (accelerated at P7, delayed at P9 and accelerated once again thereafter).

With regard to the AR pathway, at P7 we found that the unselective antagonist 8SPT had no effect on the maturation of postsynaptic clusters (*p >* 0,05, Fisher’s test Fig. 8a) although when 8SPT was applied in the period P5-P8 (observation at P9, Fig. 8b) MS2 clusters increased and MS1 and MS3 clusters decreased (*p* < 0,005), which indicates some delay in the transition from MS2 to MS3. Also, many of the few MS3 clusters remain polyinnervated with two or three axons (*p* < 0,005, Fig. 9b). Interestingly, we observed that after daily applications of 8SPT between P5 and P15 the postsynaptic maturation seems to be partially retained at the MS3 subtype (Fig. 8c). Thus, AR in normal conditions without inhibition can accelerate maturation somewhat during the P7-P9 period. Interestingly, exposure of the LAL muscle to the agonist ADO does not unambiguously change the normal distribution of the clusters at P7 (although it decreases MS3 slightly and a number of these clusters are innervated by three or more axons [*p* < 0,01, Figs. 8a and 9a]) and P9 (although there is a slight decrease in MS1, *p* < 0,01). This indicates that the tonic effect of the AR manifested by using 8SPT can not be clearly changed with exogenously added agonist. Which AR subtypes are involved in the tonic effect of endogenous ADO? We analysed the maturation of nAChR clusters after selective block of A_1_R with DPCPX or A_2A_R block with SCH-58261. Our data indicate that blocking A_1_R at P7 and both A_1_R and A_2A_R at P9 delays the maturation of normal clusters meaning that both receptor subtypes can accelerate postsynaptic maturation in normal conditions.

**Fig. 10:**
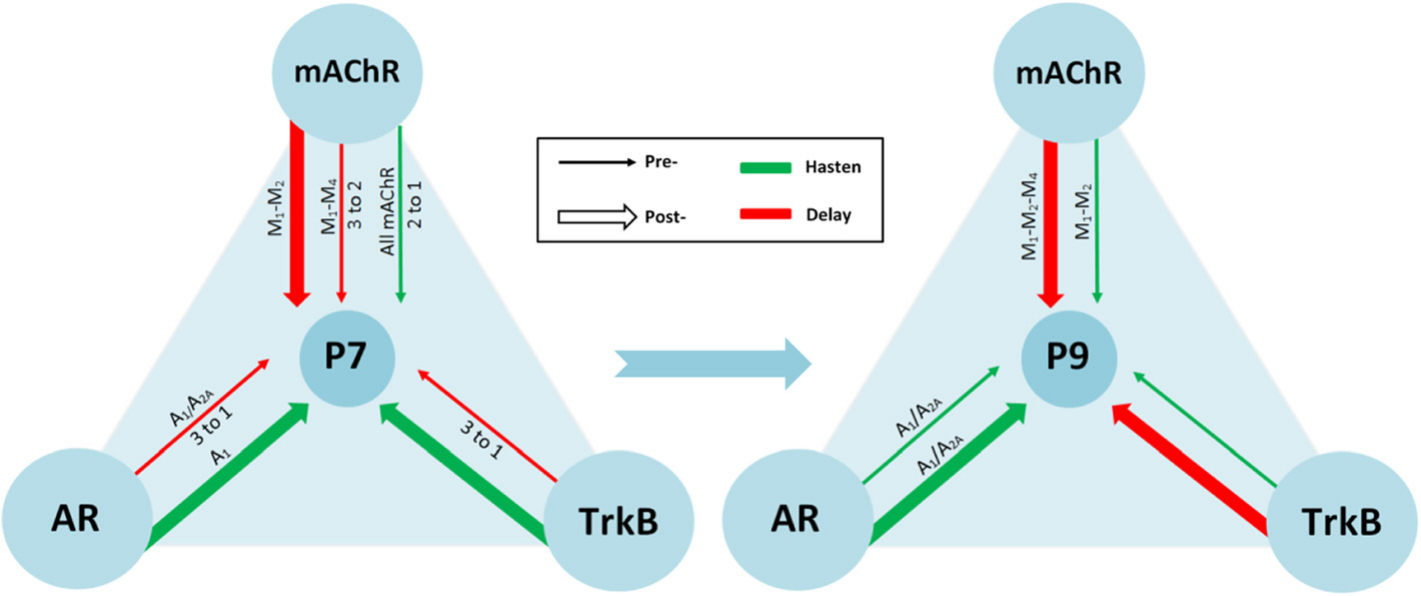
Influence of the mAChR, AR and TrkB receptors on postnatal axonal elimination and synaptic maturation

The diagram in Fig. 10 is a graphic representation of the influence of the mAChRs, and the AR and TrkB receptors on postnatal axonal elimination and postsynaptic maturation.

## Discussion

The main observation of the present study is that the coordinated action of the mAChRs (M_1_, M_2_ and M_4_), AR (A_1_R and A_2A_R) and TrkB signalling modulates the conditions of axonal competition and promotes (around P7-P9) the disconnection of supernumerary nerve endings.

### Presynaptic mAChRs M_1_-, M_2_- and M_4_-subtypes modulate axonal loss

Although there is not agreement about whether all mAChR subtypes are present in the NMJ [25, 31], some of these receptors play a role in ACh release both during development [18, 21, 32] and in the adult [32, 33]. In P6-P7 mice, we observed that M_1_ and M_4_ subtypes are involved in a mechanism that delays axonal elimination. However, the action of all muscarinic receptors as a whole indicates that the muscarinic mechanism increases the rate of axonal loss in dual junctions and, therefore, the final transition to the monoinnervation. It seems that NMJs with different maturation levels have different sensitivities to muscarinic regulation. The relative levels of these receptors or differences in turnover rate may contribute to the different effects observed. Using Western blotting we observed that in the adult, M_1_, M_2_, M_3_ and M_4_ receptors are more abundant than in the newborn [25]. In fact, changes in the expression of muscarinic receptors during development have been described in embryonic chick heart and retina [34], in carotid body, petrosal and superior cervical ganglion of the cat [35] and in rat brain [36]. In addition there are differences in the internalization and turnover of the mAChR family members [37, 38] and endocytosis may favour the coupling of the receptors to different signal transduction cascades [39].

However, the M_1_-M_2_ subtype pair (in substitution of the M_1_-M_4_ pair) cooperates to favour the full sequence of synapse elimination at P9 (the three-to-one axon transition). The delayed two-to-one transition induced by AT at P7 (which reveals accelerated axonal elimination in normal conditions without the inhibitor) may be interpreted as an early manifestation of the consistent mAChR-modulated axonal loss that is fully manifested at P9. The muscarinic mechanism appears to operate at close to maximum capacity and, therefore, may not be able to increase its efficacy beyond P7 with agonists like OXO. Interestingly, in spite of the continued presence of the M_1_ and M_2_ inhibitors, the elimination process comes to its normal conclusion at the end of the second postnatal week (P15). This suggests that other signaling mechanisms help to resolve the correct synaptic connectivity. Alternatively, M_1_ and M_2_ signaling may be not required at all for the final step of axonal elimination because the receptor inhibitors produce only transient perturbations in elimination but axon los is completed normally around P15. Our interpretation is that all considered receptors (see later) intervene in modulating the conditions of the competition between nerve endings, possibly helping to determine the winner or the lossers but, thereafter, the time and conditions of the final elimination would occur with some autonomy.

In summary, the results show that a tonic muscarinic mechanism initially delays axonal elimination (a selective M_1_-M_4_ effect). However, the overall mAChR effect may accelerate the last phase of axonal disconnection, the two-to-one transition. Thereafter the muscarinic effect at around P9 clearly promotes elimination of all supernumerary nerve terminals (an M_1_-M_2_ effect).

Which mAChR subtypes couple to regulate ACh release? In the mature NMJ, M_1_ and M_2_ mAChRs modulate evoked transmitter release by positive and negative feedbacks, respectively [13, 40]. M_2_ is more prevalent [20, 31]. During synaptogenesis, [13, 14, 41], in the monoinnervated junctions and the strong terminal in dually innervated junctions both M_1_ and M_2_ are coupled to potentiate ACh release. However, in the weakest nerve terminal in dual junctions only M_2_ potentiates release whereas M_1_ and M_4_ couple to inhibit ACh secretion. A mAChR-PKC-VDCC cascade is involved in controlling ACh release in the weak ending. Blocking PKC, VDCCs (P/Q-, N- or L-type or Ca^2+^ influx) or mAChRs (M_1_- and/or M_4_-subtypes) can lead to similar percentage increases in the size of the synaptic potentials evoked by weak axons [15–17].

How are related the release capacity of the strong and weak endings and the loss of axons described here? At P7, the release capacity of the weakest endings was increased by the inhibitors PIR and MT3, whereas ACh release from the strong ending was reduced or unaffected [16]. Thus, the difference in ACh release between the strong and weak nerve endings is reduced, and this fact may change the competitive conditions of the nerve terminals. We do not know exactly how is the ACh release capacity of the weak and strong endings in the LAL muscle at P9. However, between P7 and P9, the percentage of polyinnervated junctions changes only by about 10%. The configuration of mAChR in the monoinnervated synapses is not mature until P15 [13], which suggests that the competitive interactions between axons peak at around P9 and their release capacity is probably not very different from what it is at P7. If this is so, the reduction of the competitive advantage and disadvantage linked to ACh release of the strong and weak endings produced by PIR and the reduccion of the strength of the different axons produced by MET (MT3 does not play at P9) may considerably delay axonal loss. We found here that this is the case.

### Contribution of AR and TrkB receptor pathways

Several data suggest the involvement of other receptors. The mAChR agents alter the time course of the synapse elimination but not its final chronology. Experimental manipulations of the PKC/PKA pathways can also change the time course but not the final conclusion of synapse elimination [23, 32, 42, 43]. This indicates that different receptors with their intracellular mechanisms can be used in the process of synapse elimination.

AR are present in the motor terminals of the newborn and adult NMJs [44, 45]. These receptors can collaborate with mAChR to reduce depression during repetitive activity [44, 46, 47]. During development, low extracellular concentrations of ADO may activate both A_1_R and A_2A_R and have a facilitatory action on ACh release [48]. Our results show that mAChR and AR delay axonal loss at P7 (although mAChR accelerate the last phase of axonal disconnection) but accelerate it at P9. The results showing an additive effect between M_1_ and A_1_ or A_2A_ are an indication of the cooperation between at least these receptors.

The BDNF-TrkB pathway also plays a biphasic role. Judging from the effect of the TrkB-IgG chimera, BDNF initially delays elimination and subsequently accelerates it. Neurotrophins and their receptors in muscle and nerve are expressed in both development and adulthood [49–54]. Low doses of BDNF rapidly induce a TrkB-dependent potentiation at developing NMJs in culture. In developing muscles, BDNF increases ACh release in both the weak and strong endings at P6-P7 [55]. In addition, exogenous BDNF increases the percentage of functional polyinnervated junctions [17]. Interestingly, exogenous BDNF infusion delayed synapse elimination in the mouse LAL muscle [56] The delaying effect of the TrkB pathway on axonal elimination at P7 described here may be releated with the BDNF potentiation of the weakest endings about to be eliminated. However, blocking the TrkB receptor or neutralizing endogenous BDNF with the TrkB-IgG chimera at P7 does not affect the quantal content of the weak endings but increases release in the strong ending, which suggests that endogenous BDNF, in this developmental period, may surprisingly reduce release in the strongest ending [55]. The delaying effect of the TrkB pathway on axonal elimination at P7 may be related to the BDNF-mediated lesser release and presumed lesser competitive force of the strong axon. The TrkB pathway accelerate elimination at P9. The progressive maturation of the NMJ at P9 may change the operation conditions of the BDNF-TrkB pathway in the strongest endings resulting in more efficient competition and axonal elimination [55, 57].

### Relation between mAChR, AR and TrkB pathways

The mAChR, AR and TrkB pathways share a link mediated by the set phospholipase C (PLC)-phosphatidylinositol 4,5-bisphosphate (PIP2)-diacylglycerol (DAG)-protein kinase C (PKC), which modulates P/Q-type VDCC [40, 58]. Interestingly, PKC couples to potentiate ACh release in the adult monoinnervated NMJ, in the strong ending in developing dual junctions and in the solitary ending of the recently monoinnervated junctions at the end of developmental maturation. However, reduces release in the weakest axons in dual junctions and, therefore, PKC may be determinant in the regulation of axonal loss [18].

### Involvement of the mAChR, AR and TrkB in the maturation of nAChR clusters

mAChR [25], AR [44] and TrkB receptors [55] are present in the postsynaptic site of NMJs and are involved in organizing them [50, 59–61]. The changes we observed may be caused by the pharmacological tools directly acting on these receptors, as a side-effect of a primary effect on the axonal elimination rate or a combination of the two mechanisms. The first change in synapse elimination may be a reduction in the quantal efficacy because of a local decrease in nAChR density [62]. This postsynaptic change may begin before the overlying axon withdraws [63]. However, polyneuronal innervation decreases considerably at a time when relatively few postsynaptic nAChR are lost [10, 23]. We found here that several situations of increased axon loss or retention do not coincide with the maturation of the nAChR clústers, which suggests independent regulation. Interestingly, prolonged M_1_ and M_2_ inhibition results in a defect in postsynaptic maturation at P15. Especially, M_1_ perturbation had a strong effect. This finding suggests a requirement for M_1_ and M_2_ signaling in postsynaptic maturation and occurs when axon loss has been completed. In addition, AR block with 8SPT and TrkB pathway block with the TrkB-Fc chimera, similarly delay postsynaptic maturation at P15 (in all cases less MS4 mature nAChR clusters) indicating also the need of these signalling pathways in postsynaptic maturation. Selective nAChR-phosphorylation by PKC and PKA is one of the causes of nAChR dispersion and stability, respectively [64–66]. An activity-dependent coordinated mAChR-AR-TrkB effect on these postsynaptic kinases could be a key mechanism in NMJ maturation.

## Conclusion

Synaptic contacts are lost throughout the nervous system during both histogenesis and ageing and experience-dependent neuronal plasticity requires maintenance of newly formed synapses, while others are eliminated. We  investigate the involvement of muscarinic, purinergic and neurotrophin receptor signaling in  developmental synapse elimination. The three receptor sets intervene in modulating the conditions of the competition between nerve endings, possibly helping to determine the winner or the lossers but, thereafter, the final elimination would occur with some autonomy and independently of postsynaptic maturation.

## Methods

### Animals

Transgenic B6.Cg-Tg (Thy1-YFP)16 Jrs/J mice were used (The Jackson Laboratory). Transgenic mice express spectral variants of GFP (yellow-YFP) at high levels in motor and sensory neurons, as well as in subsets of central neurons. Axons are brightly fluorescent all the way to the terminals. No expression is detectable in nonneural cells. All experiments were conducted on Thy1-YFP-expressing mice. In some cases, we check our results with C57BL/6J mice (The Jackson Laboratory).

Experiments were performed on the *Levator auris longus* (LAL). Neonatal pups of either sex (4–30 days) were obtained and the date of birth was designated postnatal day 0 (P0). We minimized the variability in our measurements by carefully monitoring the timing of conception. Also, the weights of the individuals were within 5 % of the mean for a given day after conception. The mice were cared for in accordance with the guidelines of the European Community’s Council Directive of 24 November 1986 (86/609/EEC) for the humane treatment of laboratory animals. All experiments on animals have been reviewed and approved by the Animal Research Committee of the Universitat Rovira i Virgili (Reference number: 0233).

### Injection procedure

The newborn mice were anesthetized with 2 % tribromoethanol (0.15 ml/10 g body weight, i.p.). Under aseptic conditions, various solutions (antagonists and agonists of the considered receptors) were administered in 50 μl of sterile physiological saline or dimethyl sulfoxide (DMSO) by subcutaneous injection over the LAL external surface as described elsewhere [22]. The animals received 2, 4 or 10 injections from postnatal day 5, and the LAL muscles were studied on days 7, 9 and 15. The solutions were administered at a concentration in accordance with the previously reported biological action of the substance [14, 55, 67].

### Tissue preparation and histochemistry

Neonatal pups were given a lethal dose of 2 % tribromoethanol. Their heads were removed and fixed in 4 % paraformaldehyde for 1.5 h. After washing in phosphate-buffered saline (PBS), LAL muscles were removed and post-fixed for 45 minutes. After washing in PBS, Thy1-YFP LAL muscles were incubated in PBS containing a 1/800 dilution of 1μg/ml tetramethylrhodamine conjugated α-bungarotoxin (Molecular Probes, Eugene, OR) for 1h at room temperature.

Double immunofluorescence and confocal analysis were performed on the C57BL/6J LAL muscle. Whole mounts of LAL were processed to detect the axons with an antibody against 200-kD neurofilamentprotein and postsynaptic nicotinic acetylcholine receptors (nAChRs) with TRITC-α- BTX (Molecular Probes, Eugene, OR). Muscles were incubated overnight only with the rabbit antibody against 200-kD neurofilament (1:1,000; Sigma) in 1 % bovine serum albumin (BSA). The appropriate secondary antibody (conjugated with Alexa-fluor 488) donkey anti-rabbit (Molecular Probes) was added and incubated for 4 h. The antibody specificity was tested by incubation in the absence of primary antibody. At least three muscles were used as negative controls (not shown). Whole muscles were mounted in Mowiol with p-phenylenediamide (Sigma).

### Morphological analysis and Confocal microscopy

NMJs were analyzed using an inverted Nikon TE-2000 fluorescent microscope (Nikon, Tokyo, Japan) connected to a personal computer running image analysis software (ACT-1, Nikon). The number of axons per endplate was counted. Because of the difficulty in determining the exact number of axonal inputs for each nAChR cluster when more than two axons converged at the same synaptic site, we classified the NMJs into three groups only: junctions that were monoinnervated, doubly innervated, or innervated by three or more terminal axons. These data enabled us to calculate the “average number of axonal inputs” and the “percentage of polyneuronal innervation” for all fibers receiving two or more axons.

In PBS, DMSO control experiments or untreated animals, we determined the number of axons per endplate between days 5 to 30 and the postsynaptic nAChR cluster morphology on days 5, 7, 9, 10 and 15. Animals were injected with PBS or DMSO over the LAL muscle. The injections were performed from day 5 and the animals sacrificed on days 7, 9, and 15. No differences were found between the muscles injected or not with PBS, either in the nAChR cluster morphology or the number of axons per endplate, thus indicating that the injection procedure did not in itself induce changes in the overall morphology of the motor endplate and nerve terminals. The final concentration of DMSO in control and drug-treated preparations was 0.1% (v/v). In control experiments, this concentration of DMSO did not affect any of the parameters studied (data not shown).

To determine the effect of different treatments on the maturity of nAChR clusters at the NMJ during the period in which polyneuronal innervation is being eliminated, the maturation of the clusters was divided into four morphological stages (MS1–MS4) on the basis of criteria from previous studies of developing mammalian NMJs [23, 26, 27, 29] (Fig. 5). MS1: Uniform nAChR oval plaque with an indistinct boundary seen in the majority of NMJs at birth. A uniformly distributed porosity can be observed within this plaque. MS2: nAChR elongated oval plaque with a few hints of inhomogeneities in receptor density. The nAChRs are denser on a few narrow ridges within the plaque. MS3: An oval nAChR plaque with one or more fluorescence-free “holes.” These holes are not innervated. MS4: The oval nAChR areas have been transformed into a more mature branched pattern with a moderately convoluted external border and high and low receptor density areas. The edge of the holes usually has a high density of receptors. High-resolution confocal images were obtained with a 63x oil objective (1.4 numerical aperture) on a Nikon TE-2000 confocal microscope. Z stacks were obtained at 0.5-μm step size for depths of 20–40 μm, and additional optical sections above and below each junction were collected to ensure that the entire synapse was included.

### Statistical analysis

All NMJs visible in their entirety were scored, with a minimum of 100 per muscle. At least six muscles were studied for each age and condition examined. Fisher’s test was applied to compare percentages. The criterion for statistical significance was *p* < 0.05. The categories were scored and the counting was performed by a person with no knowledge of the age or treatment of the animals. The data are presented as mean ± SD.

### Drugs

#### Purinergic agents

##### Non-selective AR agonists and antagonists

The stock solution of adenosine 5′-triphosphate disodium salt hydrate (ADO; A9251, Sigma-Aldrich, St. Louis, MO) was made up as a 100 mM solution in deionized water. The stock solution of 8-(p-sulfophenyl)theophylline (8-SPT; A013, Sigma-Aldrich, St. Louis, MO) was made up as a 100 μM solution also in deionized water. The working solutions were adenosine (25μM) and 8-SPT (100 μM).

##### Selective A_1_ R and A_2A_ R antagonists

The stock solutions were 8-cyclopentyl-1,3-dipropylxanthine (DPCPX; C101, Sigma-Aldrich) 50 mM, and 2-(2-furanyl)-7-(2-phenylethyl)-7H-pyrazolo[4,3-e] [1, 2, 4] triazolo[1,5-c]pyrimidin-5-amine (SCH-58261; 2270, Tocris, Bristol, UK) 100 mM, both dissolved in DMSO. Working solutions were DPCPX (100 nM) and SCH-58261 (50 nM).

#### Muscarinic agents

##### Non-selective mAChR agonists and antagonists

The stock solutions were oxotremorine M (OXO; O100, Sigma - Aldrich, St. Louis, MO) 50 mM; atropine (AT; A0132, Sigma - Aldrich, St. Louis, MO) 200 μM both dissolved in deionized water. Working solutions were oxotremorine M (1μM) and atropine (2μM).

##### Selective M_1_, M_2_ and M_4_ mAChR antagonists

The stock solutions were pirenzepine dihydrochloride (PIR; 1071, Tocris Bioscience) 10 mM; methoctramine (MET; M105, Sigma – Aldrich, St. Louis, MO) 1 mM; muscarinic toxin 3 (MT-3; M-140, Alomone Labs) 50 μM. The working solutions used were pirenzepine (10 μM), methoctramine (1μM), and muscarinic toxin 3 (100 nM).

#### TrkB receptor-related agent

The following stock solutions was used: recombinant human trkB/Fc Chimera (trkB-Fc; 688-TK;R&D Systems), 100μg/ml. Working solution was trkB-Fc 5μg/ml.

## Abbreviations

A_1_R, A_1_-type receptors; A_2A_R, A_2A_-type receptors; ACh, acetylcholine; ADO, adenosine; AR, adenosine receptors; BDNF, Brain-derived neurotrophic factor; BSA, bovine serum albumin; DMSO, Dimethyl sulfoxide; LAL, Levator auris longus muscle; M_1,_ M_1_-type muscarinic acetylcholine receptor; M_2,_ M_2_-type muscarinic acetylcholine receptor ; M_4,_ M_4_-type muscarinic acetylcholine receptor; mAChR, muscarinic acetylcholine receptor; MS, maduration stage; nAChR, nicotinic acetylcholine receptor; NMJ, neuromuscular junction; NT-4, neurotrophin-4 PBS, phosphate-buffered saline; NTR, neurotrophin receptors; PKA, protein kinase A; PKC, protein kinase C; SD, Sprague-Dawley; TrkB, tropomyosin-related kinase B receptor; VDCC, voltage-dependent calcium channels
